# Topics of Public Interest

**Published:** 1916-04

**Authors:** 


### TOPICS OF PUBLIC INTEREST.

---

**War Relief Clearing House for France and Her Allies.** Contributions are needed as follows: zinc oxid adhesive plaster, 12 inches wide, operating tables, bed pans, small black rubber drains, rubber sheets, hot water bottles, syringes, thermometers, scalpels, scissors, surgical instruments, needles, hypodermic cases, clamps, rubber gloves, sheets, blankets, hospital garments. Obviously, supplies must be in usable condition. These should be sent prepaid to Ware-room of P. S. D. Fund, care of War Relief Clearing House, 133 Charlton St., N. Y., whence they will be forwarded free of charge to Paris and distributed as needed. A committee of physicians and dentists, C. N. B. Camac chairman, is endeavoring to secure pledges of $2.00 a month from the professions in this country for the relief of their medical brethren. Last month, a brief allusion to this philanthropy was published in our advertising pages, the forms of the reading matter having been closed at the time that our attention was called to the appeal by Dr. Charles Cary. We wish it distinctly understood that the policy of this journal is one of strict neutrality, that our endorsement of this appeal is based solely on humanity and professional fraternity and that the same support will be given to similar philanthropies without reference to nationality.

---

**Assistant Physicist.** The U. S. Civil Service Commission announces an examination for men only, May 3, to fill a vacancy in the Bureau of Standards at Washington. The salary is $1,400-$1,800. The degree of Ph. D. or three years of post graduate training in optics, including spectroscopy, is required and the age is limited to 25-45. In the examination, spectroscopy is given a weight of 50%, publication or thesis 10%, education, training or experience 40%. Applicants should write immediately for form 1312. Examinations will be held at numerous points in the U. S.

---

**Chief Statistician for Vital Statistics.** The U. S. Civil Service Commission announces an examination to fill a vacancy in the Bureau of the Census, Washington. The salary is $3,000. Competitors will not be assembled for examination, but will be rated as follows: Practical tests in statistics 20, thesis 20, education 25, experience 35. They must be graduates of a medical school and must have at least 4 years' experience in charge of the vital statistics of a city or state, or must have had some similar position. Applicants should apply immediately for form 1312. The first and second subjects of examination must be prepared on special paper to be furnished and must be in the hands of the commission by May 16.

**Maximum Natural Temperature.** Frederick Monsen, the explorer, while conducting observations for the U. S. Government in Death Valley noted Aug. 14, 1891, a temperature of 128 F. in the shade and 165 in the sun. The following year he noted unofficially, respective readings of 137 and 171. These are said to be the maxima for the world. Death Valley is one of the few regions of the world below sea level, the stream entering it disappearing by evaporation. The more or less marshy termination of this stream contains pretty sharply demarcated deposits of sodium chlorid, sodium borate and sodium nitrate. The humidity in the summer is too slight to be measured even by accurate hygrometers. 2-4 gallons of water daily must be drunk to maintain life; in fact, a supply must be carried and used frequently. The urine is scant and the high amount of moisture eliminated mainly by the skin does not show as sensible perspiration. Horses require 20 gallons of water a day.

**Dietetic Department of University of Rochester.** The late Lewis R. Ross former president of the Board of Trustees, who had suffered from dyspepsia, has left $750,000 to the University to establish a Dietetic Dept. This represents approximately 2/3 of his estate. Yale, Cornell and Columbia already have similar departments.

**Narcotic Law Decision.** Dr. David Cohen of Buffalo was indicted for violation of Section 248 of the Public Health Law in "prescribing heroin, morphine and other dangerous drugs and not keeping a record thereof." He was defended by A. L. Harrison, Esq., Counsel for the Medical Society of the County of Erie, the trial being before Judge Charles A. Wheeler, Feb. 26. Mch. 3, Judge Wheeler handed down his decision that the words prescribing and dispensing were not synonymous; that a physician may prescribe these drugs to a patient after examination and need not keep a record thereof; that only when a physician dispenses, acting as a druggist, must he keep a record of the drug he dispenses. This is a most important decision.

**The Degraff Memorial Hospital** of N. Tonawanda, has received $10,000 by the will of the late James Sweeney Thompson, president of the hospital board, to equip an X-ray laboratory.

---

**A Bill to establish a Bureau for the Study of the Criminal, Pauper and Defective Classes** has been introduced into both houses of congress. The salary of the director is set at $3,000, with $6,800 for a clerical staff. In our opinion, the job is scarcely worth the agitation required to get the bureau started. One of the great obstacles to preparedness for war and efficiency in peace is the spending of money in small amounts on an unnecessary duplication of energy, by the various branches of the government.

---

**Removal of Stains.** (Abstracted from various sources). Brush, shake or remove by vacuum cleaner, loose dust from all fabrics before attempting to remove stains.

Grease and oils of animal or vegetable nature (Glycerole in combination with an alkali to form soap and glycerin). Woolen clothes, sponge with weak ammonia. Do not use fixed alkali. Linen and cotton, soak in weak ammonia water. Soap suds may also be used, but not too strong. Silks, benzene, soaking if possible, as sponging leaves a ring on evaporating. Or chalk or kaolin may be used to absorb the grease. Mineral oils, vaseline, etc. Lay a piece of blotting paper or coarse brown paper over the spot and hold a moderately hot iron over it. The paper will absorb the grease. Several pieces of paper may be necessary.

Sugar, syrup, glue, mucilage, blood. Wash with an abundance of water, changing frequently. For blood, use cool water immediately as it is very difficult to remove an old stain.

Varnish and paint. Linen, cotton and woolen, turpentine or benzene, preferably turpentine before the spot dries. Silk, benzene and soap, rubbing gently. Old spots should be softened with chloroform.

Vegetable colors, red wine, fruit, aniline ink, etc. Linen, vapor of burning sulphur or weak chlorin water, for certain dyes, alcohol containing a few drops of hydrochloric acid. Cotton, woolen and silk, dilute tartaric acid. Obviously, without knowing the exact nature of the coloring matter, it is impossible to give a rule of universal application, and dyes in the goods may also be removed.

Iron rust, iron ink. Linen, warm oxalic acid solution. Cotton and woolen, repeated washing with dilute solutions of citric acid. These may also remove dyes, and silk is especially hard to clean.

Tar, wagon grease and auto grease. Linen, soap and turpentine, alternating with water under pressure. For iron spots (black) remaining, try oxalic acid. Cotton and woolen, rub hard with lard, soak well, wash alternately with water and turpentine. Silk, use benzene instead of turpentine, use water under pressure.

Stains are removed from the skin as follows: Silver nitrate, use ammonia water immediately, before blackening (reduction to metallic silver) occurs. Remove black silver deposits with solution of potassium cyanid or potassium iodid. The yellow stain remaining may be removed by a hyposulphite solution.

Chrysarobin. Chloroform or alcohol.

Resorcin. Citric acid solution.

Picric acid. Potassium sulphite solution, one minute, followed by soap and water.

Pyrogallic acid. No method found practical.

Potassium permanganate. Oxalic acid solution.

Anilin dyes, as from typewriter ribbon, indelible pencil and certain stains used in the laboratory. Alcohol, plus a few drops of hydrochloric or nitric acid, applied on a piece of cotton.

Gram stain. Alcohol applied on cotton.

Auto grease, etc. Gasoline applied on cotton, followed by washing with sapolio or soap containing some detergent.

---

**Columbia College of Dentistry.** Plans have been made for a department of dentistry, the requirements for admission and first two years of the course being identical with those of the College of Physicians and Surgeons. At the end of the second year, the dental students will receive special instruction. This will be the second dental school forming a department of a university, in the state, the first being that of the University of Buffalo. The course at Columbia will be four years in all.

---

**A Bill to Standardize the Treatment of Tuberculosis** in the United States, to provide Federal aid in caring for indigent tuberculous persons, and for other purposes, has been introduced by Wm. Kent of California. Be it enacted by the senate and house of representatives of the United States of America in congress assembled, That within the appropriations made from time to time for such purposes the secretary of the treasury is hereby authorized to aid state authorities in providing care and treatment for indigent tuberculous persons who are citizens of the United States, but not legal residents of the states in which they are temporarily located, and for this purpose may designate such public or private hospitals and sanatoria as may be necessary. Prior to being designated to receive patients, and from time to time, said institutions shall be subject to inspection by officers of the public health service in order to determine the facilities and methods available and in use for care and treatment of patients, and the secretary of the treasury is further authorized to prescribe standards to which institutions shall conform in order to obtain the benefits of this act.

Sec. 2. That hospitals and sanatoria designated in accordance with the provisions of this act shall be entitled to and may receive from the federal treasury a subvention fixed annually by the secretary of the treasury, but not exceeding 75 cents per diem for each indigent patient admitted with the approval of the secretary of the treasury: Provided, that the state in which said indigent tuberculous patient is admitted to a hospital or sanatorium for treatment shall pay or cause to be paid a subvention, not less than that paid by the federal government, toward the cost of caring for such patient in said hospital or sanatorium. Subventions under this law will be granted only in the case of indigent patients who have submitted satisfactory evidence that they were not assisted by any person or institution to leave their legal residence or did not themselves leave in order to receive benefits under this act.

Sec. 3. That the secretary of the treasury is authorized to issue regulations governing the designation of institutions and establishment of standards and for otherwise carrying out the provisions of this act.; and he is further authorized to collect and make available for general use information and descriptive matter relative to the construction, equipment and maintenance of hospitals, sanatoria, and similar institutions.

Sec. 4 That detailed estimates of the sums required annually to carry out the provisions of this act shall be submitted hereafter in the usual book of estimates.

---

**Government Manufacture of Munitions of War.** Hon. Clyde H. Tavenner, representative from Illinois, in two speeches, "The Navy League Unmasked" and "The World Wide War Trust," raises an important issue regarding preparedness, which he does not by any means oppose. He does not especially emphasize the ethical and diplomatic problems arising from the sale of munitions by private firms, but holds that the government is paying 20-60% more than if it carried on its own manufactures, even under shorter hours and higher wages, and objects to the exploitation by private firms of the skill of officers trained at government expense, and of the results of experiment carried on directly at the expense of the government.

**The National Guard** at Niagara Falls will give rudimentary instruction in military drill to classes of citizens unable to enlist—a policy which we have advocated with special regard to the needs of physicians.

---

**Commission Government.** In January, 1915, we discussed this matter, then recently decided upon for Buffalo, calling special attention to the need of economic administration. The first tax rate under this form of government will be $28 per thousand, the highest on record. The same excuse is offered as under the former system, that the preceding administration made an artificially low tax rate—which was not noticeably low but about the highest on record—but the real reason may be found that nearly every candidate for election as commissioner put the soft pedal on economy and the loud pedal on civic improvement and municipal philanthropy.

---

**Change in City Physicians for Buffalo.** In place of 10 city physicians at small salaries, supplemental to private practice, four appointments have been made for full time work at $1800 a year. These appointments are provisional pending the establishment of a civil service list. Dr. A. C. Callahan, West Side District, 1067 Grant Street; Dr. Leon S. Kurek, North Broadway, Central and Stock Yards District, 591 William Street; Dr. John H. Wild, Kensington and Sycamore District, 770 East Ferry Street; Dr. Charles Leone, Oak, Chicago, Cazenovia and Water Front District, 404 Seneca Street. Office hours 8:30-10, 1-3, 7-8. The Superintendent of the Poor and the police may call upon the city physicians for services to the needy.

---

**The Bath Hospital** opened its new home, formerly the residence of the late Dr. A. H. Cruttenden, March 4. It will now accommodate 35 patients, being the largest hospital between Rochester and Elmira. The hospital began four years ago in the old Pultney Land Office. Recognizing the need of better accommodations, Dr. Cruttenden provided by will that his residence should become the property of the hospital. The equipment has been provided largely by the generosity of Drs. H. J. Wynkoop and D. H. Smith, but an open-door policy will be maintained toward the profession.

---

**Standards for Bottled Waters.** The Bureau of Chemistry of the U. S. Dept. of Agriculture has established the following: Total number of bacteria developing on standard agar plates incubated 24 hours at 37 degrees C., not more than 100 per c. c., estimated from averages of not less than two plates showing such numbers and distribution of colonies as to indicate that the estimate is accurate. Not more than one out of five 10 c. c. portions shall show organisms of colon bacillus group, following technic of U. S. Public Health Service. (Note: The policy of this journal has been not to accept advertisements of bottled waters without reliable bacteriologic testimony as to purity).

---

**The Child Welfare Board** held its annual weighing day in the Tonawanda High School March 4, and in North Tonawanda March 7. Drs. C. W. Clendenan, R. H. Wilson, W. W. Britt and T. P. C. Bernard were in charge.

---

**Proper Food for Young Children.** Farmers' Bulletin No. 717, by the U. S. Dept. of Agriculture, prepared by Caroline L. Hunt under the direction of Dr. C. F. Longworthy, is a non-technical discussion containing recipes and direction for mothers. Clean whole milk, at least a quart a day is the basis of the diet for a child 3-6 years old, plus bread and other cereals, butter and other fats, vegetables and fruits and "simple sweets." This can be obtained by physicians, and is of special value in directing the care of children at a distance from milk stations and from other special sources of supplies.

---

**Automobile Statistics.** 234,032 motor vehicles were registered for N. Y. State in 1915, an increase of 64,000 over 1914. This represents about one car to 40 persons. For Erie Co. the registration was 18,471, about 1:30. The average price for the state was $814 as opposed to $2,123 in 1907. Western N. Y. has more automobiles in proportion to population than any other comparable area in the world.

---

**The Harrington Lectures** May 30-June 1, at the commencement of the University of Buffalo, will be given by Dr. Milton J. Rosenau, Prof. of Preventive Medicine and Hygiene at Harvard. Two lectures will be on Anaphylaxis and one on Education for Public Health Service as a Career.

---

**College Merger.** The College of Physicians and Surgeons and the Medical School of the University of Maryland, both located in Baltimore, have been merged.

---

**The Central and Northern Alumni Assn. of the University of Buffalo** held its second annual dinner in Syracuse March 9. 125 graduates of various departments were present.

---

**Old Age Poverty.** S. W. Strauss of Chicago, in Leslie's Magazine, states that 66% of persons dying in the U. S. leave no estate whatever, that only 9% leave more than $5,000, and that the average for the other 25% is only $1,300. It is further stated that 98% of the population of the U. S. live from day to day on their wages. On reflection, these statistics are not so bad as they appear. Almost one-third of all deaths occur in persons under 20, very few of whom could have accumulated anything except by inheritance or gift. Thus, of adults, not quite half leave no estate, and about 14% leave $5,000 or more, while about 39% average $1,300. Many small estates of persons who have not only supported themselves but their families, with a little left over, are settled informally, and many large estates are undervalued. 100%of the population living on incomes, is a reductio ad absurdum which shows that a close approach to a hand-to-mouth existence for the majority of adults really means general prosperity. It must be remembered also that a large number of persons, especially elderly men and women, that appear to be dependents, really earn their living in the households of relatives, and could do so, though less agreeably, among strangers if necessary. However, these qualifications must not be understood as deprecating thrift nor such social and economic conditions as tend to a more equable distribution of wealth.

